# The salivary microbiota of patients with acute lower respiratory tract infection–A multicenter cohort study

**DOI:** 10.1371/journal.pone.0290062

**Published:** 2024-01-11

**Authors:** Matthew B. Rogers, Ashley Harner, Megan Buhay, Brian Firek, Barbara Methé, Alison Morris, Octavia M. Peck Palmer, Susan B. Promes, Robert L. Sherwin, Lauren Southerland, Alexandre R. Vieira, Sachin Yende, Michael J. Morowitz, David T. Huang

**Affiliations:** 1 University of Pittsburgh, Pittsburgh, Pennsylvania, United States of America; 2 Pennsylvania State University, State College, Pennsylvania, United States of America; 3 Wayne State University, Detroit, Michigan, United States of America; 4 The Ohio State University Wexner Medical Center, Columbus, Ohio, United States of America; 5 Children’s Hospital of Pittsburgh, Pittsburgh, Pennsylvania, United States of America; Children’s National Hospital, George Washington University, UNITED STATES

## Abstract

The human microbiome contributes to health and disease, but the oral microbiota is understudied relative to the gut microbiota. The salivary microbiota is easily accessible, underexplored, and may provide insight into response to infections. We sought to determine the composition, association with clinical features, and heterogeneity of the salivary microbiota in patients with acute lower respiratory tract infection (LRTI). We conducted a multicenter prospective cohort study of 147 adults with acute LRTI presenting to the emergency department of seven hospitals in three states (Pennsylvania, Michigan, and Ohio) between May 2017 and November 2018. Salivary samples were collected in the emergency department, at days 2–5 if hospitalized, and at day 30, as well as fecal samples if patients were willing. We compared salivary microbiota profiles from patients to those of healthy adult volunteers by sequencing and analyzing bacterial 16-rRNA. Compared to healthy volunteers, the salivary microbiota of patients with LRTI was highly distinct and strongly enriched with intestinal anaerobes such as *Bacteroidaceae*, *Ruminococcaceae*, and *Lachnospiraceae* (e.g., mean 10% relative abundance of *Bacteroides* vs < 1% in healthy volunteers). Within the LRTI population, COPD exacerbation was associated with altered salivary microbiota composition compared to other LRTI conditions. The largest determinant of microbiota variation within the LRTI population was geography (city in which the hospital was located).

## Introduction

The human microbiota consists of a diverse and abundant community of microbial cells dwelling within each individual. Recent data suggest connections between perturbations of the respiratory microbiota and respiratory disease. Studies in this area have examined chronic respiratory illnesses in outpatients, such as asthma and idiopathic pulmonary fibrosis, or critically ill patients receiving mechanical ventilation [1–5]. However, there are limited data on patients presenting to the emergency department (ED) with acute lower respiratory tract infection (LRTI). Dysbiosis of the upper respiratory tract microbiota has been found in patients presenting to the ED with pneumonia [6], and a preponderance of a *Haemophilus* species has been reported in patients with chronic lung conditions hospitalized with pneumonia [7]. Acute LRTI is a common cause of ED visits, incurs a significant healthcare burden, and is an important patient population to investigate. Profiling their microbiota could provide real time, actionable, and personalized information that could be used to improve clinical outcomes for LRTI patients.

Within the oropharynx and airway, most published studies have examined sputum samples or tracheal aspirates, but the salivary microbiota is mostly underexplored [8]. Prior studies of saliva have been limited by sample size and single center design, and it is unclear whether the salivary microbiome varies with acute disease, patient characteristics, and geography [9–13] This knowledge gap is potentially important because saliva represents an easily accessible source for investigation and the development of diagnostic tools [14,15]. Further, past studies have indicated that the lung microbiota, which cannot be studied noninvasively, overlaps with the oral microbiota [16]. Therefore it is possible that analysis of saliva could provide important information about the lung microbiota [17], and in turn could provide information about LRTI etiology. We therefore sought to explore in a prospective multicenter cohort, the composition of the salivary microbiota in patients presenting with clinically diagnosed acute LRTI, association with baseline clinical features and outcomes, and heterogeneity across geographic regions.

## Materials and methods

We conducted an investigator-initiated, multicenter prospective cohort study of adult patients with suspected acute LRTI presenting to the EDs of seven hospitals in southwestern and central Pennsylvania, southern Michigan, and central Ohio between May 2017 and November 2018. We collected a longitudinal series of saliva samples and corresponding fecal samples and associated clinical data to microbiota analyses as described below. The University of Pittsburgh and all site institutional review boards approved the protocol. Thermo Fisher Scientific provided an unrestricted grant to fund the study, received enrollment updates and reviewed a manuscript draft, but had no role in study design or manuscript writing.

### Study subjects

Eligible participants were 18 years or older and had a clinical diagnosis in the ED of acute LRTI (< 28 days duration). For broad applicability we sought to enroll routine cases of uncomplicated LRTI commonly seen in the ED. We therefore excluded patients receiving antibiotics prior to ED presentation, on chronic dialysis, receiving vasopressors or mechanical ventilation in the ED, with known severe immunosuppression, with accompanying non-respiratory infection, known empyema, or metastatic cancer, or who had surgery in the past seven days, were incarcerated, homeless, or previously enrolled in this study within the past 30 days. Fecal samples were obtained by self-swabbing and patients could opt out of providing fecal samples.

We categorized the initial diagnosis of LRTI into final diagnoses of acute exacerbation of chronic obstructive pulmonary disease (COPD), asthma exacerbation, acute bronchitis, community-acquired pneumonia, and other, using the classification schema of a recent large LRTI trial [18]. This cohort was enrolled prior to the SARS-Cov-2 pandemic.

### Clinical procedures

We asked participants to provide a saliva sample and a fecal sample (or a rectal swab if the patient preferred) at three timepoints—in the ED, on days 2–5 if hospitalized, and on day 30. If a patient was still hospitalized at day 30, we obtained samples in the hospital. Site coordinators sent these locally obtained samples to the University of Pittsburgh. For patients discharged from the ED or hospitalized and discharged prior to day 30, we provided discharge sample collection kits with instructions, pre-labeled sample collection supplies, and a prepaid, pre-addressed return envelope to the University of Pittsburgh. Samples were collected into Zymoresearch DNA/RNA Shield Collection tubes to avoid growth of bacteria while samples were transported at room temperature.

To encourage participant compliance with obtaining and mailing the day 30 samples and to gather post-discharge health status information, we contacted patients by phone at days 15 and 30. We compensated participants for each timepoint swabs that were obtained (ED, day 2–5, day 30), provided a study calendar with reminders for the phone calls and day 30 sample collection, required sites to collect multiple phone contacts and to notify us of patient discharge dates within 48 hours of discharge, and provided a text messaging option for patients who preferred texting versus phone calls. For saliva collection, subjects were instructed to refrain from using mouth wash, brushing their teeth, or flossing for 12 hours before sample collection. Samples were not stored for more than 20 days at ambient temperature, and all samples were shipped within 20 days of collection. Each center was sent highly detailed collection protocols in order to standardize collection methods. **S1 Appendix** contains illustrated details of saliva sample collection procedures. **S2 Appendix** details procedures for fecal sample collection. **S3 Appendix** is an inventory of the collection kit and contains further information on storage and shipping of samples.

All experimental protocols were approved by the University of Pittsburgh Institutional Review Board (STUDY20070139: MAPLE) and were carried out according to these guidelines. All patients or their authorized representatives provided written informed consent and indicated willingness to provide salivary samples at all pre-defined study timepoints.

We obtained clinical data via chart review by site research staff, and phone calls at days 15 and 30 by coordinating center staff. For adverse outcomes, we used the composite outcome used in a recent large trial [18] of endotracheal intubation, administration of vasopressors, renal failure, lung abscess/empyema, development of pneumonia in patients presenting with non-pneumonia LRTI, rehospitalization or death.

#### Healthy reference population of individuals without LRTI

For a reference group of individuals without LRTI, we used sequencing data from saliva samples collected from 290 patients without chronic health conditions enrolled in the Dental Registry and DNA Repository (DRDR) from the School of Dental Medicine at the University of Pittsburgh [19]. In brief, this project has the approval of the University of Pittsburgh Institutional Review Board and all participants provided written informed consent. Biospecimens were linked to patients’ electronic health record (EHR) data, permitting analysis of associations between oral microbiota data obtained from DNA extracted from the specimens with dental and medical conditions. All data were de-identified, and biospecimens were linked to EHRs using a unique study number rather than personal identifying information. The microbiotas of these patients were then sequenced at the Center for Medicine and the Microbiome (CMM) at the University of Pittsburgh (https://www.microbiome.pitt.edu). Henceforth we refer to the DRDR cohort as the ‘healthy’ group, and the study cohort as the LRTI group. To adjust for the effect of age on microbiota composition we excluded 13 DRDR patients under 18 years old, leaving 276 subjects / samples in our analysis.

### Microbiome procedures

#### DNA extraction

DNA from LRTI patient samples was extracted using the Qiagen DNeasy PowerSoil HTP DNA Isolation kit. Briefly, for the saliva, fecal or rectal swab samples, the swab heads were cut off directly into individual wells of the bead plate and stored overnight at -80°C. The next day the Bead Solution and Solution C1 were added, and the plates were incubated at 65°C for 10 minutes in a bead bath. The homogenization step was performed on a Retsch Oscillating Mill MM400 with 96-well plate adaptors for 7.5 minutes at a frequency of 20Hz. The plates were rotated 180° and the cycle was repeated. The Solution C2 and C3 steps were combined (200 μl of each added) to improve DNA yield. All remaining steps followed the manufacturer’s centrifugation protocol.

DNA from the DRDR (healthy) samples were brought to room temperature and centrifuged for 5 minutes at 10,000xg. After the supernatant was removed 1 ml of extraction buffer (10mM Tris-HCL pH 7.8, 5mM EDTA, 0.5% SDS) was added to the buccal cell pellet and thoroughly mixed. A 5ul solution of proteinase K was added and the samples were incubated in a 56°C water bath overnight. After removal, the samples were vortexed and 500 ul of 10M Ammonium Acetate was added. Samples were inverted for 3 minutes and centrifuged at 21,000xg for 15 minutes. The supernatant was then transferred to two eppendorfs with an equal volume of cold isopropanol and shaken vigorously. Samples were then incubated at -20°C for a minimum of 30 minutes, after which they were centrifuged at 10,000xg for 20 minutes at 4°C. The supernatant was poured off and 1 ml of cold 70% EtOH was added, and samples were inverted 3 times. Samples were then centrifuged at 10,000xg for 5 minutes at 4°C. The supernatant was poured off and samples were allowed to air dry before 100 ul of TE buffer was added. Samples were kept in a 4°C refrigerator for 2–3 days before ensuring that the DNA was completely dissolved by reading the concentrations of the samples.

#### 16S-rRNA gene amplicon PCR and sequencing

PCR amplification of the small subunit ribosomal RNA gene (16S-rRNA) was performed in triplicate 25 μl reactions. Amplicons were produced utilizing primers adapted for the Illumina MiSeq. Amplicons target the V4 region and primers utilized either the Illumina adapter, primer pad and linker (forward primer) or Illumina adaptor, Golay barcode, primer pad and linker (reverse primer) followed by a sequence targeting a conserved region of the bacterial 16S-rRNA gene as described by Caporaso, et al [20]. Five extraction controls were sequenced alongside the experimental samples.

#### Microbiota analysis

Paired-end amplicon sequence reads were demultiplexed using QIIME2 package [21]. The first 22 bps of the reads were trimmed, and both forward and reverse reads were truncated at 180 bp at which point we observed a decline in the average phred score of randomly sampled reads. The DADA2 [22] algorithm, executed within the QIIME2 package [21] was used to predict amplicon sequence variants (ASVs). ASVs were aligned using MAFFT [23], and a phylogenetic tree was generated using Fasttree. Finally a naive-bayes feature classifier was trained on the V4 region of 16-rRNA using the Greengenes database (v 13.5). Taxonomic assignment was then carried out on the ASV sequences using the feature classifier. ASV table, taxonomy table and phylogenetic tree were then loaded into R using the Phyloseq [24] import_biom function. Samples with amplicon counts less than 4,000 were removed from the analysis. The five extraction controls included in the sample preparation were searched against the Greengenes (13.5) database using BlastN [25]. Most of the extraction controls (4/5) had less than 10 reads. Only one had a read-pair matching, which matched an unknown bacterium distantly related to *Veillonella*. We could not find an ASV matching this amplicon in the rest of the samples. One extraction control contained 860 paired end reads. Along with common soil bacteria, this sample also included an abundance of reads matching human gut bacteria *Bacteroidaceae* and *Enterobacteriaceae*. This control was only associated with 3 patients and 5 samples. We treated these samples as contaminated and removed them from downstream analysis.

To adjust for the well documented effects of age on microbiota composition we excluded DRDR patients that were below the lower range of LRTI patients (18 years old; 13 patient samples).

Alpha diversity and beta diversity calculations were done using both Vegan [26] and Phyloseq [24]. Alpha diversity in its simplest form accounts for the richness of different ASVs within a single environment (for example, the number of bacterial species in a saliva sample from an individual patient). A more informative alpha diversity metric is the Shannon index, which quantifies not only ASV richness, but also distribution of ASV abundances. High values of Shannon diversity indicate a high ASV richness and even distribution of ASVs. Beta diversity accounts for differences between environments (for example, differences in microbial species abundance and phylogenetic distance between samples from different patients). Beta diversity is typically measured as distances between samples based on abundances of ASVs and may also include phylogenetic distances (Unifrac metric).

All statistical analyses of the microbiota data were done in R using the libraries Vegan [26], Phyloseq [24], ANCOM [27], ANCOM-BC [28] and LOCOM [29]. We wrote additional custom code for comparisons against the healthy samples. All code, ASV table, tree, ASV sequences, phylogenetic tree, control sequence data and metadata are available at https://github.com/MattRogers2/.

#### Statistical analyses

To compare the composition of the salivary microbiota in our LRTI study group versus healthy controls, we collected saliva samples from all timepoints, and compared alpha diversities (Shannon metric) using a Wilcoxon rank-sum test. We calculated beta diversity distances between samples using the weighted-unifrac metric [30] implemented in the Phyloseq R library [24], then observed whether samples clustered as discrete groups using principal coordinate analysis (PCoA), and tested the significance of variance between these group differences using the Permutational Multivariate Analysis of Variance test (ADONIS2) encoded in the vegan R library. As some subjects had samples taken at up to three time points we stratified the ADONIS2 permutations for each time point to adjust for multiple samples per subject. Due to significant differences in age differences in the LRTI and healthy cohorts, we included age as an interacting term in the ADONIS2 analysis comparing cohorts. Taxonomic composition was compared between groups using the Analysis of Composition of Microbiomes (ANCOM) [27] R package, Analysis of Composition of Microbiomes with Bias Correction (ANCOM-BC) [28] and logistic compositional analysis (LOCOM) [29].

We looked for differences in the salivary microbiota profiles associated with a composite endpoint of adverse outcomes within 30 days of enrollment, including hospital re-admission, development of pneumonia in a patient without pneumonia at enrollment, empyema, organ failure, endotracheal intubation, vasopressor requirement, renal failure, and death [18].

Due to a limited number of subjects with samples beyond a baseline sample, we chose to focus our comparisons on samples taken at the baseline timepoint for clinical variable comparisons. We performed a multivariable ADONIS2 analysis of 24 categorical variables taken from patient demographics and clinical features. We conducted a separate ADONIS2 analysis including the five LRTI diagnoses. ADONIS2 analyses were repeated while stratifying permutations on city in which the hospitals were located. To examine heterogeneity of the salivary microbiota across cities, we performed PCoA and pairwise ADONIS2 comparisons on the hospital city locations. We also performed multiple chi-squared analyses between clinical variables and the hospital location city to verify independence of patient clinical features from hospital location. Finally, a correlogram was generated from patient metadata and outcomes using the Pearson metric and the R corrplot library. For all statistical tests, we rejected null hypotheses above a p-value of 0.01.

## Results and discussion

### Results

#### Clinical cohort

We enrolled 150 patients with 3 requesting withdrawal, for a final analysis cohort of 147 patients. Patients were middle age (mean 51 years), 61.9% female, and 60.5% Black; 30.6% received antibiotics in the ED and 47.6% were admitted to hospital (**Table 1**). For saliva samples, 145 patients (98.6%) provided samples in the ED, 48 of the 70 patients admitted to hospital (68.6%) provided a day 2–5 sample, and 50 patients (34.0%) provided a day 30 sample. For fecal samples, 39 patients (26.5%) provided samples in the ED, 21 of the 70 patients admitted to hospital (32.9%) provided a day 2–5 sample, and 50 patients (34.0%) provided a day 30 sample. Fourteen patients experienced an adverse outcome within 30 days of enrollment. The mean age of the 290 patients from the healthy cohort was 35 years old, with a male to female ratio of 1.6:1.

**Table 1:** Clinical and demographic data collected from subjects participating in both the LRTI study and DRDR studies.

Characteristics	Total (n = 147)		Healthy (n = 290)	Healthy > 18 yrs old (n = 277)
Age–yr	51 ± 18.2		35±15.5	36.3± 15.1
Male sex–no. (%)	56 (38.1%)		142 (49%)	137 (49.4%)
**Race/ethnicity–no. (%)**				
White	56 (38.1%)		212(73%)	208 (75.0%)
Black	89 (60.5%)		42(14.4%)	36 (12.9%)
Hispanic	3 (2.0%)		5(1.7%)	4 (1.4%)
Asian	0		23(7.9%)	21 (7.5%)
Other/Undisclosed	0		8(2.7%)	8 (2.8%)
**Comorbidities§**				
Charlson comorbidity score	1.4 ± 1.2			
Current smoker–no. (%)	55 (37.4%)			
COPD	65 (44.2%)			
Asthma	67 (45.6%)			
**Home medications–no. (%)** ^**‡**^				
Home oxygen	18 (12.2%)			
Oral corticosteroids	28 (19.0%)			
Inhaled corticosteroids	46 (31.3%)			
Inhaled long-acting bronchodilators	41 (27.9%)			
Leukotriene receptor antagonists	11 (7.5%)			
**Symptoms**				
Duration—days	4 ± 4.9			
Cough–no. (%)	107 (72.8%)			
Dyspnea–no. (%)	126 (85.7%)			
Sputum production–no. (%)	63 (42.9%)			
Chest discomfort–no. (%)	73 (49.7%)			
Chills–no. (%)	46 (31.3%)			
**Clinical findings**				
Temperature (degrees Celsius)	36.9 ± 0.5			
Heart rate	90.0 ± 14.8			
Respiratory rate	20.0 ± 4.6			
Mean arterial pressure (mmHg)	96.2 ± 13.7			
Oxygen saturation (%)	96.0 ± 3.1			
Rhonchi—no. (%)	17 (11.6%)			
Wheezing—no. (%)	74 (50.3%)			
**Final diagnoses—no. (%)** ^**††**^				
Asthma	67 (45.6%)			
COPD	63 (42.9%)			
Acute bronchitis	22 (15.0%)			
CAP	29 (19.7%)			
Other	6 (4.1%)			
Hospitalized–no. (%)^	70 (47.6%)			

COPD—chronic obstructive pulmonary disease; CAP—community-acquired pneumonia.

* Plus-minus values are means ± SD.

‡ Home medications defined as medications taken by patient in last 7 days.

†† 12 patients had CAP and COPD, 10 patients had CAP and asthma.

^ Mean length of stay was 5.2 days (SD 4.9).

#### Microbiota composition of saliva samples from study subjects differ from healthy samples in diversity and taxonomic composition

**Fig 1:**
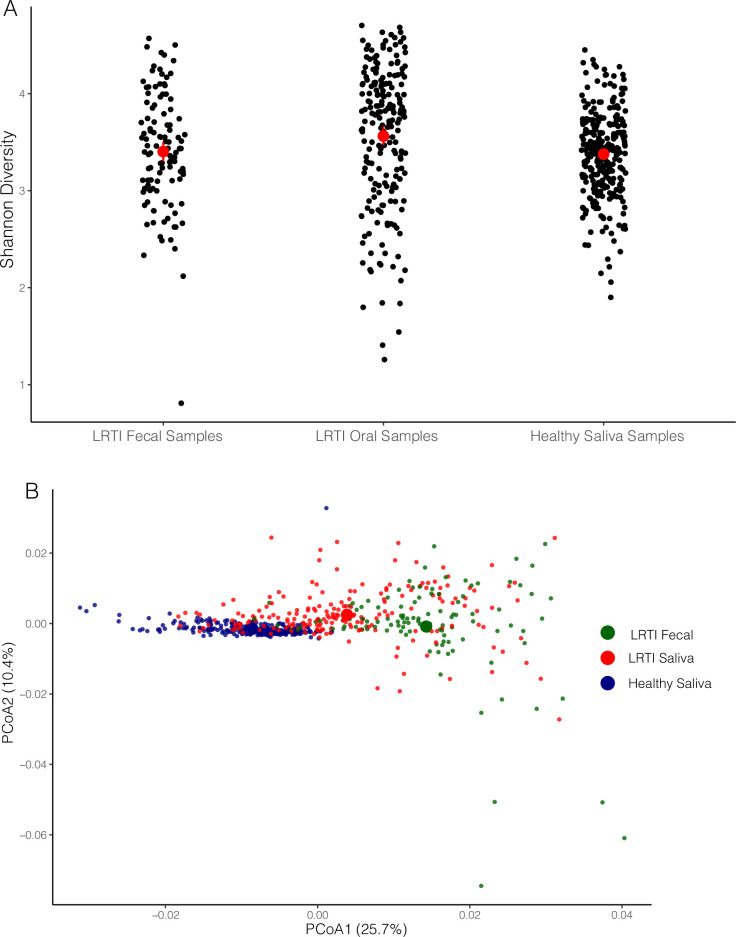
Alpha diversity and beta diversity plots. (A) Scatterplot of Alpha diversity values (Shannon metric) across groups. Mean values for each group are shown as a central red dot with non-parametric confidence limits indicated by red lines. (B) PCoA plot of weighted unifrac beta diversity distances between LRTI saliva samples, LRTI fecal samples and healthy saliva samples. Large circles indicate centroid of each group.

Alpha diversity was significantly higher in saliva samples from patients with LRTI relative to saliva from healthy volunteers (mean 3.56 for LRTI saliva samples vs 3.38 for healthy saliva, p-value < 0.01) (**Fig 1A**). Saliva and fecal samples from study subjects and saliva samples from healthy volunteers each formed a discrete cluster (**Fig 1B)**. Differences between all groups were significant (ADONIS2, R^2^ = 0.164, **S1B Table**). Age was a significant, albeit weak contributor to variation between cohorts (R^2^ = 0.008). The greatest difference between body site / cohorts was found between LRTI fecal samples and healthy saliva samples (R^2^ = 0. 202, p-value < 0.001). As expected, variation between LRTI samples and healthy samples was also considerable (R^2^ = 0.126, p-value < 0.001). Relative to variation between saliva samples from the two cohorts, variation between LRTI fecal samples and LRTI saliva samples was unexpectedly lower than expected (R^2^ = 0.056, p-value < 0.001). Statistics for these analyses are shown in **S1A**, **S1B** and **S1C Table**.

**Fig 2:**
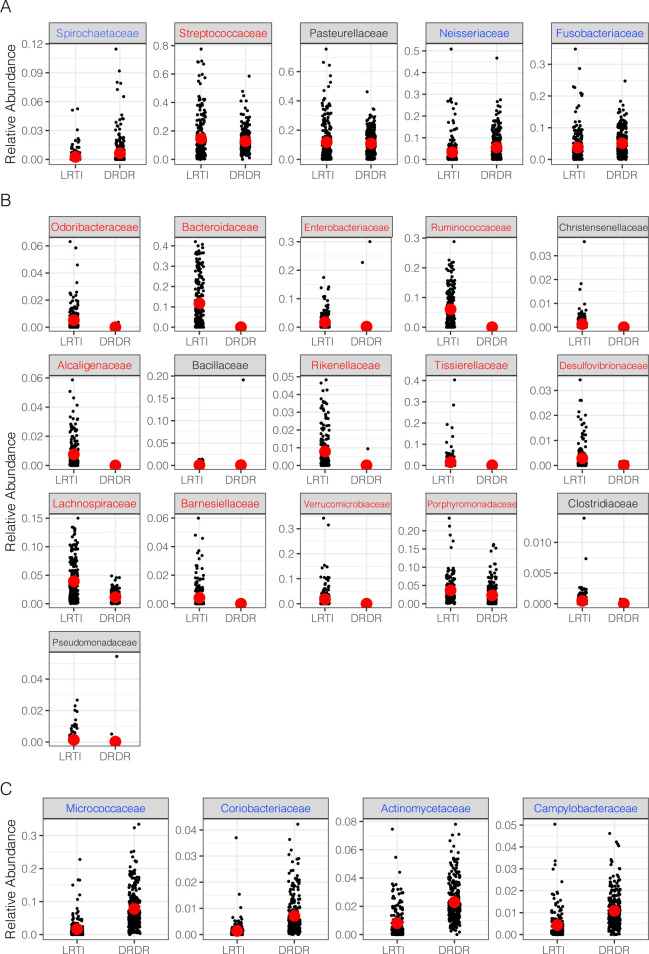
Taxonomic composition of saliva samples by cohort and body site. A) Common bacterial families by relative abundance shared between saliva samples from the healthy cohort and the LRTI cohort. B) Differentially abundant families predicted by ANCOM that are enriched in the LRTI group versus the healthy group. C) Equivalent to (B) but with families that are enriched in the healthy group. Taxa with red labels are predicted to be enriched in the LRTI group by LOCOM, while those with blue font are predicted to be depleted by LOCOM.

The taxonomic composition of most saliva samples from both cohorts largely resembled published profiles of the typical saliva microbiota, with members of *Pasteurella*, *Spirochaetes* and *Streptococcus* abundantly represented (**Fig 2A**). However, ANCOM also identified multiple taxonomic families that were differentially abundant between the healthy and LRTI saliva samples. Saliva samples from LRTI study subjects were enriched with prominent members of the normal colonic microbiota, notably the *Bacteroidaceae*, *Ruminococcaceae* and *Verrucomicrobiaceae* families (**Fig 2B**). Each taxon was relatively abundant in LRTI patient samples, but virtually absent in the healthy group. LRTI saliva samples were also enriched in the family *Alcaligenaceae* (which contains pathogens such as *Bordetella* species) and were depleted of common saliva commensals such as *Micrococcaceae* (**Fig 2C**).

Analysis of taxonomic composition using LOCOM broadly agreed with the ANCOM results (S1 Fig) but some taxa that appeared to be of similar abundance were predicted to be depleted in LOCOM (see blue text in Figs 2A and S1).

#### Association of clinical features and outcome with the salivary microbiota

Multivariable analysis of baseline variables revealed that the variable with the greatest effect size was the city from which the sample was taken (R^2^ = 0.154, p-value = 0.001; **S1D and S1E Table**). This finding will be discussed further below. As a result of this unexpected variation in beta-diversity between city location, we stratified the ADONIS2 analysis on city of sampling variable to remove confounding effects that city location may have (**S1F and S1G Table**).

**Fig 3:**
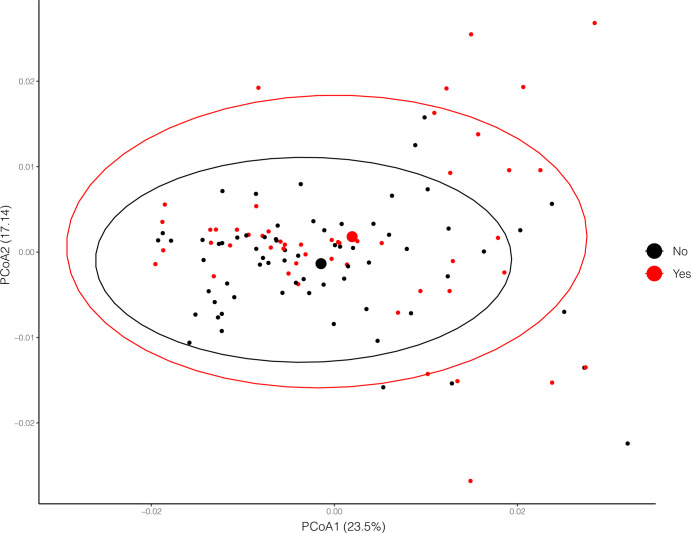
PCoA plots of baseline saliva samples by clinical variables. PcoA plot of samples from subjects experiencing a COPD exacerbation, red points indicate a positive diagnosis of COPD exacerbation. COPD exacerbation was the only clinical finding that was predicted to be significantly different by ADONIS multivariable analysis (p-value < 0.01).

In the ADONIS2 analysis stratified on hospital city, we found significant variation between the salivary microbiota of baseline samples from subjects that had an LRTI classification of COPD exacerbation (R^2^ = 0.019, **S1G Table, Fig 3**). Overall, each of these variables had a minimal effect size relative to the effect of the city in which the hospitals were located. We did not find a significant difference in the salivary microbiota of patients who experienced an adverse outcome and patients who did not (R^2^ = 0.010, p-value = 0.171)

We performed a similar ADONIS2 analysis on baseline fecal samples using the same clinical and demographic variables. Unlike the baseline saliva samples we did not observe a significant difference in geographic location despite a modest effect size (R^2^ = 0.128, p-value = 0.0397). This finding may also have been due to a limited number of fecal samples (34 samples), with most (21/34) collected in Pittsburgh hospitals. Unlike saliva samples, we found no significant variation in the fecal microbiota of baseline samples when grouped by outcome or clinical/demographic variables (**S1H and S1I Table)**.

**Fig 4:**
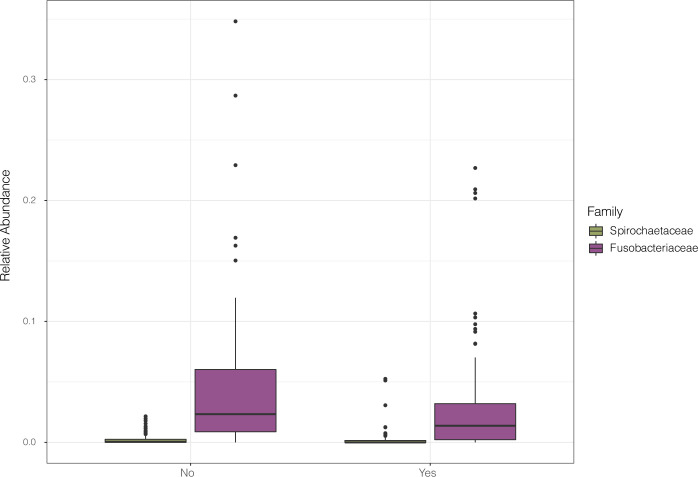
Variations in taxonomic composition by clinical variable. Differences in taxonomic family composition between patients that experienced an outcome of COPD exacerbation and those that did not. City of hospital site was used as a co-variate to correct for taxonomic families that varied between hospital sites. Differentially abundant families are represented as boxplot graphs, coloured by taxon, shown in the shared legend. The COPD exacerbation diagnosis is displayed on the x-axis, and relative abundances are plotted along the y-axis.

ANCOM predicted only minor differences in taxonomic variation in saliva samples from a patient with COPD exacerbation after adjusting for hospital city location (**Fig 4**). In samples from subjects with COPD exacerbation, ANCOM predicted a reduced abundance of oral commensals from the families *Fusobacteriaceae* and *Spirochaetaceae*.

#### Variation of salivary microbiota by geographic location

**Fig 5:**
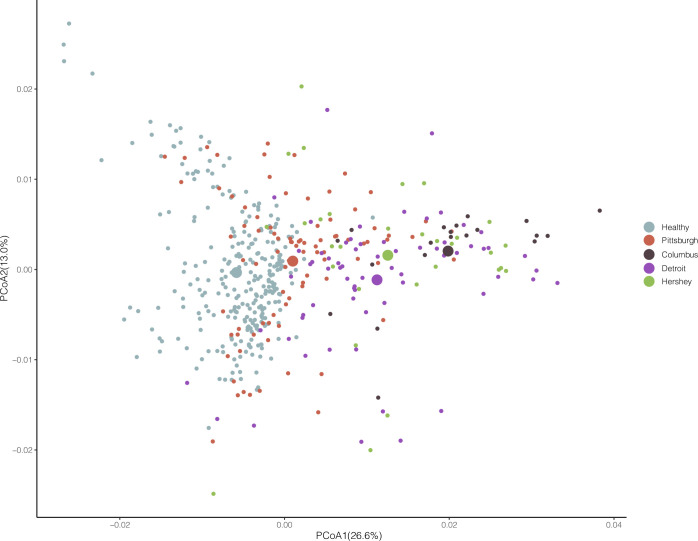
PCoA plot of Weighted Unifrac distances between all saliva samples. Samples are coloured by city, each point indicates an individual sample, large points indicate the position the centroid of each hospital site group.

The variable with the greatest effect size in our comparisons of initial salivary samples within the LRTI group was the city in which the sample was collected (ADONIS2; P-value<0.001, R^2^ = 0.185). (**S1D Table and Fig 5**). Multiple pairwise ADONIS2 tests showed differences between the healthy group and all other LRTI city groups were significant at p-values < 0.001. Similarly, differences between the Pittsburgh hospitals and all other sites were significant at p-values < 0.001(S1J Table), and pairwise comparisons comparisons between all cities except Hershey vs. Detroit were significant. In addition to differences in beta-diversity between sites, we also found that Shannon alpha diversity of the Pittsburgh samples was significantly lower than the other sites, though not when compared against the healthy group. (Wilcoxon rank-sum test, p-value <0.01) (results not shown).

The microbiota composition of samples from each city compared against Pittsburgh samples also varied. Saliva samples from Pittsburgh consistently had more oral commensals, including bacteria from the *Streptococcaceae*, *Micrococcaceae* and *Leptotrichiaceae* families. In contrast, samples from Pittsburgh hospitals had a comparative absence of gut-associated bacteria (*Ruminococcaceae*, *Lachnospiraceae*, *Bacteroidaceae*) compared against those from other cities (**S2 Fig)**, though Pittsburgh LRTI patient saliva samples were still enriched with gut associated bacteria compared to healthy controls (**S3 Fig).**

Clinical features of the patient populations also varied across cities (**S1K Table**). A total of eight clinical or demographic features failed a chi-squared test of independence when tested against city location. Most prominently of these was race of the subject (chi-squared test, p-adjusted = 6.22e-07), with only 5% white patients in Detroit, compared to 46% in Pittsburgh, and 87% in Hershey. A correlogram of clinical metadata (**S4 Fig**) indicated that patient metadata positively correlated with Pittsburgh as a collection city included chills, cough and a LRTI classification of community associated pneumonia or acute bronchitis. Inversely correlated was the recent use of oral or inhaled corticosteroids and steroids administered in the ED. Taken together these data indicate that patients in Pittsburgh suffered a higher rate of CAP and bronchitis. Conversely clinical features from patients in Detroit included significantly higher use of oral corticosteroid use, a history of asthma and a LRTI classification of exacerbation of asthma.

## Discussion

We found that the salivary microbiota of patients presenting to the ED with acute LRTI had unique characteristics compared to healthy controls, clinical features were associated with the salivary microbiota, and that the salivary microbiota varied significantly by geography.

The high abundance of gut microbiota in the salivary microbiota of LRTI subjects was the most notable finding, and similar to recent findings of gut flora within the lung microbiota of patients with acute respiratory distress syndrome [31]. It has been suggested that this shift in microbiota results from translocation during acute illness [32]. Past studies have generally examined the lower respiratory tract rather than saliva, but one study examining the salivary microbiota in respiratory disorders also observed a high abundance of intestinal taxa (*Lachnospiraceae*) in salivary samples from patients with chronic COPD [12]. These findings are notable as a core microbiome principle is site specificity, meaning that microbial communities from different body sites, such as the mouth, lungs, and gastrointestinal tract, are generally distinct in a healthy state. An alternative explanation could be that lung microbiota dysbiosis predisposes an individual to an LRTI. A final possibility is that there may be a bi-directional relationship between LRTI and dysbiosis. Of note, salivary and oral microbiota dysbiosis have been found associated with not only respiratory disorders [33], but also multiple systemic disorders [34], including head and neck cancer [35], autoimmune disorders [36], and Alzheimer’s disease [37], and these relationships continue to be explored. With further study on greater numbers of individuals over extended periods of time, it may be possible to establish the presence of gut microbiota in saliva as a marker of respiratory disorders [38]. Future longitudinal studies on patients with LRTI may help shed some light on directionality of the dysbiosis / LRTI relationship.

We also found that patients who experienced a clinical outcome of COPD exacerbation were significantly different in terms of beta-diversity, though with a low effect size. Taken together, these findings provide proof of concept that salivary microbiota can differ with clinical features.

Samples from patients with a classification of COPD exacerbation also had a slight reduction in oral commensal families, including *Fusobacteriaceae* and *Spirochaetaceae*. *Fusobacteriaceae* and *Spirochaetaceae* are both contributors to dental caries and oral plaque formation, but are also highly prevalent members of the oral microbiota [39,40]. One previous study has also shown the Spirochaete genus *Treponema* to be enriched in healthy individuals compared against individuals with COPD [41].

The largest determinant of the salivary microbiota was the geographic location of the hospital. Similar results with fecal samples have been published and our finding is consistent with past studies demonstrating the geographic impact on microbiota composition [42]. Taxonomic composition was also noted to vary between cities, with the composition of samples from Pittsburgh most closely ressembling a healthy oral microbiota, though still enriched in gut bacteria when contrasted with the healthy cohort (also located in Pittsburgh). Other large studies with samples collected across multiple centers have not observed geographic variation in the salivary microbiota [43]. Site variation in sample collection and processing may have contributed to these differences, though we provided standardized instructions across sites. Finally, it is highly possible that city specific differences in subject populations are contributing to apparent differences in geography. Regardless, it is important to consider the geographic location of study participants when designing microbiota studies because a positive result from a single-center study may not be generalizable to other populations.

### Limitations

Gut microbiota in saliva samples can indicate contamination during processing and sequencing of samples, but the absence of gut flora in saliva samples from the healthy subjects argues against this possibility. Further, past studies have indicated that extraction methodology has little impact on the composition of sampled salivary microbiota [44]. Less than ten percent of the cohort experienced an adverse outcome, limiting our ability to associate the salivary microbiota with an adverse outcome. Finally, we note that there is a significant difference in the mean and age ranges of the healthy subjects compared against the LRTI patients. Further studies on the salivary microbiome of healthy individuals will be required to determine if our observations of an enrichment of gut microbiota in the salivary microbiome of LRTI patients are the result of a difference in ages between the healthy and LRTI group.

In conclusion, we found the salivary microbiota of patients with acute LRTI differed from healthy controls mainly due to an enrichment with bacteria typically associated with gut flora. We further found a large degree of variation occurred between cities in which the samples were collected and that patients with a LRTI classification of COPD exacerbation differed significantly from other patients with LRTI. These findings offer preliminary insights into future investigations of the salivary microbiota in acute illness. With further knowledge of the immune role played by the host in LRTI associated dysbiosis and a thorough metagenomic analysis of bacterial pathways future investigations could determine how to best use an easily accessible biospecimen to classify respiratory infection, understand pathophysiology, and identify potentially clinically actionable biomarkers of infection.

## Supporting information

S1 Fig Scatter plot of differentially abundant taxa between LRTI group and control group predicted by LOCOM method. Taxonomic families appear along the and points are distributed along the X-axis according to effect size. Size of point indicates mean relative abundance of each family.
(DOCX)

S2 Fig Volcano plot of differentially abundant taxa between Pittsburgh baseline saliva samples and those of other cities. Taxa predicted by ANCOM-BC to be either enriched in other cities compared against Pittsburgh. Taxa enriched in the above cities appear to the right of the dotted line and vary along the x-axis according to their log-fold difference in abundance (those to the right are higher in the compared city; those to the left are higher in Pittsburgh). The y-axis shows the -log10 (FDR) value, taxa above 0.05 significance are labelled. Blue and red shading indicate whether the family is typical is the oral or gut microbiota respectively.
(DOCX)

S3 Fig Volcano plot of differentially abundant taxa between the healthy cohort and baseline saliva samples from the Pittsburgh LRTI cohort and those of other cities. Taxa enriched in the above cities appear to the right of the dotted line and vary along the x-axis according to their log-fold difference in abundance (those to the right are higher in the compared city; those to the left are higher in the healthy cohort). The y-axis shows the -log10 (FDR)value, taxa above 0.05 significance are labelled. Blue and red shading indicate whether the family is typical is the oral or gut microbiota, respectively.
(DOCX)

S4 Fig Correlogram of patient metadata and LRTI types showing relationships between variables. **Size of circles indicate strength of correlation by R2 value.** Shading of circle also indicates strength of correlation by R2 value, blue shading indicates correlation, red shading indicates inverse correlation. Variables are clustered by R2 value using hclust. Only associations with a p-value < 0.05 (Pearson metric) are shown.
(DOCX)

S1 Table Results of statistical tests. (DOCX)

S1 Appendix Illustrated procedures for collecting saliva samples. (DOCX)

S2 Appendix Illustrated procedures for collecting fecal samples. (DOCX)

S3 Appendix Inventory of each collection kit and information on storage and shipping of samples. (DOCX)

## Abbreviations

CAP: Community acquired pneumonia
COPD: Chronic obstructive pulmonary disease
ED: Emergency Department
LRTI: Lower respiratory tract infection
ASV: Amplicon Sequence Variant
PCoA: Principal Coordinates Analysis
FDR: False discovery rate
