# Correction: The salivary microbiota of patients with acute lower respiratory tract infection–A multicenter cohort study

**DOI:** 10.1371/journal.pone.0319276

**Published:** 2025-02-07

**Authors:** Matthew B. Rogers, Ashley Harner, Megan Buhay, Brian Firek, Barbara Methé, Alison Morris, Octavia M. Peck Palmer, Susan B. Promes, Robert L. Sherwin, Lauren Southerland, Alexandre R. Vieira, Sachin Yende, Michael J. Morowitz, David T. Huang

The Supporting Information files (S1–S4 Figs, S1 Table, and S1–S3 Appendices) are uploaded incorrectly. Please view the correct S1–S4 Figs, S1 Table, and S1–S3 Appendices below.

## Supporting information

S1 Fig Scatter plot of differentially abundant taxa between LRTI group and control group predicted by LOCOM method. Taxonomic families appear along the and points are distributed along the X-axis according to effect size. Size of point indicates mean relative abundance of each family.
(EPS)

S2 Fig Volcano plot of differentially abundant taxa between Pittsburgh baseline saliva samples and those of other cities. Taxa predicted by ANCOM-BC to be either enriched in other cities compared against Pittsburgh. Taxa enriched in the above cities appear to the right of the dotted line and vary along the x-axis according to their log-fold difference in abundance (those to the right are higher in the compared city; those to the left are higher in Pittsburgh). The y-axis shows the -log10 (FDR) value, taxa above 0.05 significance are labelled. Blue and red shading indicate whether the family is typical is the oral or gut microbiota respectively.
(EPS)

S3 Fig Volcano plot of differentially abundant taxa between the healthy cohort and baseline saliva samples from the Pittsburgh LRTI cohort and those of other cities. Taxa enriched in the above cities appear to the right of the dotted line and vary along the x-axis according to their log-fold difference in abundance (those to the right are higher in the compared city; those to the left are higher in the healthy cohort). The y-axis shows the -log10 (FDR)value, taxa above 0.05 significance are labelled. Blue and red shading indicate whether the family is typical is the oral or gut microbiota, respectively.
(EPS)

S4 Fig Correlogram of patient metadata and LRTI types showing relationships between variables. Size of circles indicate strength of correlation by R2 value. **Shading of circle also indicates strength of correlation by R2 value, blue shading indicates correlation, red shading indicates inverse correlation. Variables are clustered by R2 value using hclust. Only associations with a p-value < 0.05 (Pearson metric) are shown.**
(EPS)

S1 Table Results of statistical tests. (XLSX)

S1 Appendix Illustrated procedures for collecting saliva samples. (PDF)

S2 Appendix Illustrated procedures for collecting fecal samples. (PDF)

S3 Appendix Inventory of each collection kit and information on storage and shipping of samples. (DOCX)
