# Supplementary material for: The salivary microbiota of patients with acute lower respiratory tract infection–A multicenter cohort study
Source: PLoS One. 2024 Jan 11;19(1):e0290062. doi: 10.1371/journal.pone.0290062 (PMC10783762; doi:10.1371/journal.pone.0290062)
Supplement: S1 Fig — Taxonomic families appear along the and points are distributed along the X-axis according to effect size. Size of point indicates mean relative abundance of each family. (DOCX) [file pone.0290062.s001.docx]

**S1 Fig. Scatter plot of differentially abundant taxa between LRTI group and Control group predicted by LOCOM method.** Taxonomic families appear along the and points are distributed along the X-axis according to effect size. Size of point indicates mean relative abundance of each family.
