# Supplementary material for: The salivary microbiota of patients with acute lower respiratory tract infection–A multicenter cohort study
Source: PLoS One. 2024 Jan 11;19(1):e0290062. doi: 10.1371/journal.pone.0290062 (PMC10783762; doi:10.1371/journal.pone.0290062)
Supplement: S2 Fig — Taxa predicted by ANCOM-BC to be either enriched in other cities compared against Pittsburgh. Taxa enriched in the above cities appear to the right of the dotted line and vary along the x-axis according to their log-fold difference in abundance (those to the right are higher in the compared city; those to the left are higher in Pittsburgh). The y-axis shows the -log10 (FDR) value, taxa above 0.05 significance are labelled. Blue and red shading indicate whether the family is typical is the oral or gut microbiota respectively. (DOCX) [file pone.0290062.s002.docx]

**S2 Fig.** **Volcano plot of differentially abundant taxa between Pittsburgh baseline saliva samples and those of other cities.** Taxa predicted by ANCOM-BC to be either enriched in other cities compared against Pittsburgh. Taxa enriched in the above cities appear to the right of the dotted line and vary along the x-axis according to their log-fold difference in abundance (those to the right are higher in the compared city; those to the left are higher in Pittsburgh). The y-axis shows the -log10 (FDR) value, taxa above 0.05 significance are labelled. Blue and red shading indicate whether the family is typical is the oral or gut microbiota respectively.
