# Supplementary material for: The salivary microbiota of patients with acute lower respiratory tract infection–A multicenter cohort study
Source: PLoS One. 2024 Jan 11;19(1):e0290062. doi: 10.1371/journal.pone.0290062 (PMC10783762; doi:10.1371/journal.pone.0290062)
Supplement: S3 Fig — Taxa enriched in the above cities appear to the right of the dotted line and vary along the x-axis according to their log-fold difference in abundance (those to the right are higher in the compared city; those to the left are higher in the healthy cohort). The y-axis shows the -log10 (FDR)value, taxa above 0.05 significance are labelled. Blue and red shading indicate whether the family is typical is the oral or gut microbiota, respectively. (DOCX) [file pone.0290062.s003.docx]

**S3 Fig.** **Volcano plot of differentially abundant taxa between the healthy cohort and baseline saliva samples from the Pittsburgh LRTI cohort and those of other cities.** Taxa enriched in the above cities appear to the right of the dotted line and vary along the x-axis according to their log-fold difference in abundance (those to the right are higher in the compared city; those to the left are higher in the healthy cohort). The y-axis shows the -log10 (FDR)value, taxa above 0.05 significance are labelled. Blue and red shading indicate whether the family is typical is the oral or gut microbiota, respectively.
