# Supplementary material for: The salivary microbiota of patients with acute lower respiratory tract infection–A multicenter cohort study
Source: PLoS One. 2024 Jan 11;19(1):e0290062. doi: 10.1371/journal.pone.0290062 (PMC10783762; doi:10.1371/journal.pone.0290062)
Supplement: S4 Fig — Size of circles indicate strength of correlation by R2 value. Shading of circle also indicates strength of correlation by R2 value, blue shading indicates correlation, red shading indicates inverse correlation. Variables are clustered by R2 value using hclust. Only associations with a p-value < 0.05 (Pearson metric) are shown. (DOCX) [file pone.0290062.s004.docx]

**S4 Fig. Correlogram of patient metadata and LRTI types showing relationships between variables. Size of circles indicate strength of correlation by R2 value.** Shading of circle also indicates strength of correlation by R2 value, blue shading indicates correlation, red shading indicates inverse correlation. Variables are clustered by R2 value using hclust. Only associations with a p-value < 0.05 (Pearson metric) are shown.
