# Supplementary material for: The salivary microbiota of patients with acute lower respiratory tract infection–A multicenter cohort study
Source: PLoS One. 2024 Jan 11;19(1):e0290062. doi: 10.1371/journal.pone.0290062 (PMC10783762; doi:10.1371/journal.pone.0290062)
Supplement: S1 Table — (DOCX) [file pone.0290062.s005.docx]

**Table S1**

(A) P-values from pairwise-Wilcoxon-test comparison of alpha-diversity between saliva samples from patients with LRTI, healthy saliva samples and fecal samples from patients with LRTI. (B) Adonis2 analysis of beta diversity distances based on specimen type including age as a covariate. (C) Pairwise-Adonis2 comparisons of each sample type combination. (D) Adonis2 multivariate comparison using only baseline saliva samples from LRTI subjects. (E) Adonis2 multivariate comparison of LRTI outcomes using only baseline saliva samples from LRTI subjects, including hospital city as variable. (F) Adonis2 multivariate comparison using only baseline saliva samples from LRTI subjects with analysis stratified on city. (G) Adonis2 multivariate comparison of LRTI outcomes using only baseline saliva samples from LRTI subjects with analysis stratified on hospital city. (H) Adonis2 multivariate comparison using baseline fecal samples from LRTI subjects. (I) Adonis2 multivariate comparison of LRTI outcomes using baseline fecal samples from LRTI subjects. (J) Pairwise-Adonis2 comparison of beta diversity distances between cities, including healthy cohort. (K) Variables that fail chi-squared test of independence compared against city. Shown are proportion of positive variables by city, and adjusted p-value for each variable.
