# Supplementary material for: The salivary microbiota of patients with acute lower respiratory tract infection–A multicenter cohort study
Source: PLoS One. 2024 Jan 11;19(1):e0290062. doi: 10.1371/journal.pone.0290062 (PMC10783762; doi:10.1371/journal.pone.0290062)
Supplement: S2 Appendix — (DOCX) [file pone.0290062.s007.docx]

**Appendix S2** Illustrated protocol for collection of patient fecal samples.
