# Supplementary material for: The salivary microbiota of patients with acute lower respiratory tract infection–A multicenter cohort study
Source: PLoS One. 2024 Jan 11;19(1):e0290062. doi: 10.1371/journal.pone.0290062 (PMC10783762; doi:10.1371/journal.pone.0290062)
Supplement: S3 Appendix — (DOCX) [file pone.0290062.s008.docx]

**Appendix S3** Inventory of collection kits with detailed instructions on shipping and storage of samples.
