# Supplementary material for: Correction: The salivary microbiota of patients with acute lower respiratory tract infection–A multicenter cohort study
Source: PLoS One. 2025 Feb 7;20(2):e0319276. doi: 10.1371/journal.pone.0319276 (PMC11805346; doi:10.1371/journal.pone.0319276)
Supplement: S2 Appendix — (PDF) [file pone.0319276.s007.pdf]

FECAL – Instructions for Collecting Specimen

BEFORE YOU GIVE A SAMPLE

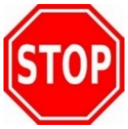

Check the collection date on your calendar and on the kit bag.
Do not use the kit until the date listed.

You will collect a fecal sample from used toilet paper.
You do **not** need to swab anywhere on your body.

INSTRUCTIONS

1. Put on the gloves provided.
2. Peel open the swab package and remove the swab, touching only the plastic handle. Do not let the tip of the cotton swab touch your fingers or any other surface.
3. Collect a small amount of feces by swabbing your used toilet paper. Fecal matter should be visible on the swab tip.
4. Open the collection tube. **Do not dump the liquid out of the tube.**
5. Place the swab tip in the solution inside.
6. Break the plastic handle of the swab in half, leaving just the swab tip in the collection tube. Throw away the plastic handle.
7. Screw the cap on the tube.
8. Invert (turn upside down) the tube 3 or 4 times.
9. Place the sealed tube in the Ziploc bag with an absorbent pad. **Do not remove the absorbent pad.**
10. Close the Ziploc bag.

AFTER YOU COLLECT SAMPLE

Once you have completed both the saliva AND the fecal swab:

1. Make sure both tubes are in the Ziploc bag.
2. Put the Ziploc bag with the samples in the pre-labeled shipping envelope we have provided.
3. Mail the shipping envelope using standard United States Postal Service.

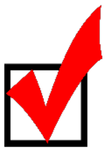

You're done! Once we receive your sample, we will reload your pre-paid debit card with $25. Please allow 6-8 week for receipt and processing.
