# Supplementary material for: Correction: The salivary microbiota of patients with acute lower respiratory tract infection–A multicenter cohort study
Source: PLoS One. 2025 Feb 7;20(2):e0319276. doi: 10.1371/journal.pone.0319276 (PMC11805346; doi:10.1371/journal.pone.0319276)
Supplement: S3 Appendix — (DOCX) [file pone.0319276.s008.docx]

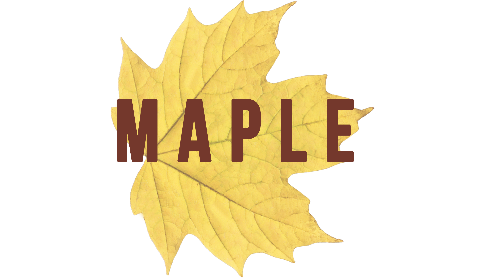
**MAPLE**

**List of Contents**

Site Name:_______________________________

**This shipment contains a total of 15 Patient Specimen Collection Kits . Each patient kit will contain 2 in-hospital kits and one 30-day in-home kit.**

| **In hospital kits (00hr and 24hr)**   - *2 swab collection tubes* - *1 biohazard bag* - *1 Ziploc bag with absorbent pad* - *Specimen ID labels (00hr and 24hr)* | **In-home Kits (*30-Day)***   - *2 swab collection tubes* - *1 Ziploc bag with absorbent pad* - *Gloves* - *Return shipping envelope* - *Patient collection SOPs for Fecal and Saliva* - *Patient FAQ* |
| --- | --- |

- **Shipping Supplies:**
  - Padded Shipping Envelopes
- **Documents:**
  - Shipping SOP
  - Overview and Procedure SOP
  - Detailed Specimen Collection SOP’s
  - Sample Packing Lists
  - Lab Supply Request forms

**Request additional kits/supplies using the Laboratory Supplies Request Form**

**Questions:**

Contact Meghan Allwes ([allwesmb@upmc.edu](mailto:allwesmb@upmc.edu)), Lauren Porter ([porterln2@upmc.edu](mailto:porterln2@upmc.edu)), Vanessa Jackson ([jacksonvm@upmc.edu](mailto:jacksonvm@upmc.edu)),


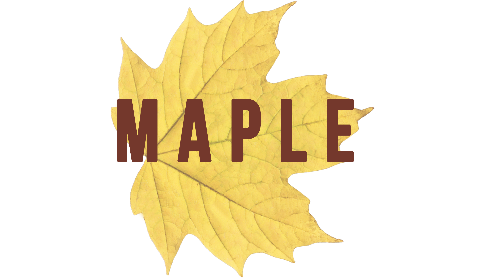
**MAPLE**

**Overview and Procedure SOP**

Each kit contains 2 in-hospital (00hr &24hr) kits and one 30-day in-home kit for the patient

**IMPORTANT: Check that the subject/specimen ID’s match the subject you are enrolling and that the 00hr and 24hr timepoints match the 30-day kit that you give to the patient**

1. **Collect in-hospital specimens (00hr and 24hr)**

- Collect one fecal swab and one saliva swab from patient for *each* timepoint
- Timepoint 1: 00hr
- Timepoint 2: 24hr
- Place provided specimen labels on tubes
- Store tubes at ambient temperature until shipped to the CRISMA lab (ship the 15^th^ and 30^th^ day of the month)
- **IMPORTANT: Specimens can only be stored for 20 days at ambient temperature and must be shipped to CRISMA lab prior to reaching 20 days ambient.**


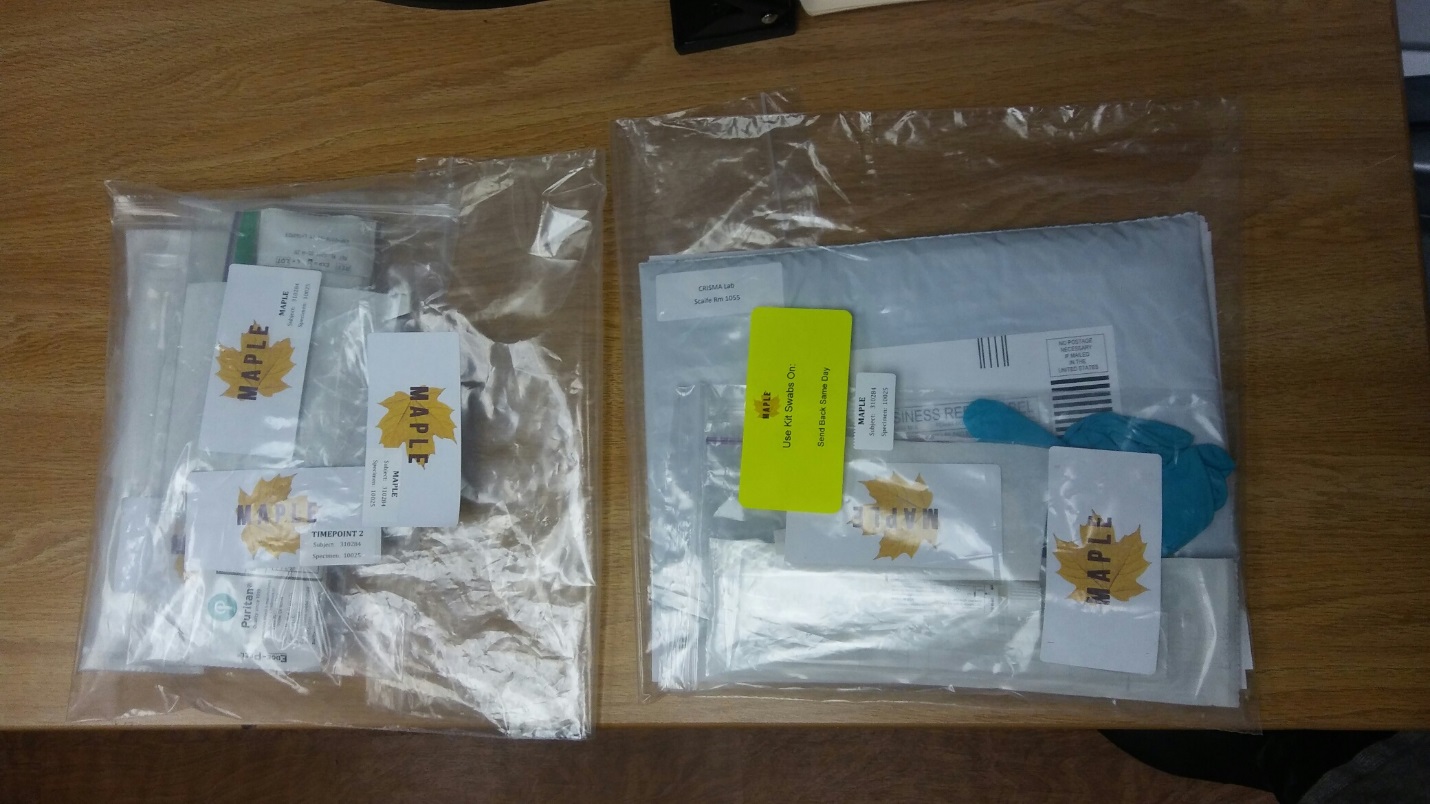


1. **Prepare in-home (30-day) kit for patient prior to discharge**

- Write the patient’s 30-day collection date on the YELLOW label on the front of their kit
- Explain the contents of the kits to patients
  - Discuss in-home collection procedure SOP’s that are included in their kit package
  - Explain that collection kit can be used on day 30 or shortly after day 30, but not before day 30
- Give patient in-home kit prior to discharge


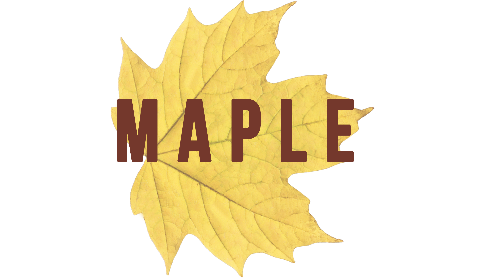
**MAPLE**

**Shipping SOP**

**IMPORTANT: Specimens can only be stored for 20 days at ambient temperature and must be shipped to CRISMA lab prior to reaching 20 days ambient**

**Shipping specimens to CRISMA:**

- You will ship the 00hr and 24hr specimens in batches on **approximately the 15^th^ and 30^th^ calendar day of the month**
- First virtually ship the specimens
- Fill out the Sample Packing List for the samples you are sending
-
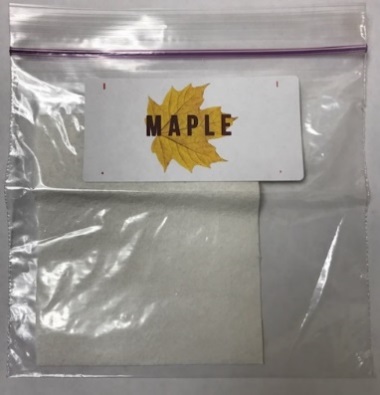
Prepare specimens for shipping:
  1. Place the labeled 00hr and 24hr specimens in the included Ziploc bags with absorbent pad (2 tubes per bag)
  2. Place Ziploc bags in the padded shipping envelope
  3. Place the Sample Packing List in the shipping envelope.
  4. Weigh padded envelope and email Lauren, Vanessa, or Sarah the shipping weight in order to obtain your shipping label
  5. Affix appropriate shipping labels to padded envelope and mail envelope to CRISMA lab


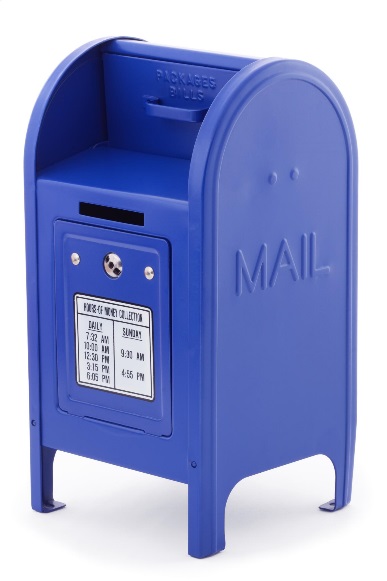


**Questions:**

Contact Meghan Allwes ([allwesmb@upmc.edu](mailto:allwesmb@upmc.edu)), Lauren Porter ([porterln2@upmc.edu](mailto:porterln2@upmc.edu)), Vanessa Jackson ([jacksonvm@upmc.edu](mailto:jacksonvm@upmc.edu)),
